# Biological and Behavioural Features of the Stenogastrinae (Hover Wasps) in a Particular Evolutionary Route to Eusociality in the Family Vespidae

**DOI:** 10.3390/insects17030322

**Published:** 2026-03-16

**Authors:** Stefano Turillazzi

**Affiliations:** Department of Biology, University of Florence, Via Madonna del Piano 6, Sesto Fiorentino, 50019 Firenze, Italy; stefano.turillazzi@unifi.it

**Keywords:** evolution of eusociality, Vespidae, Stenogastrinae, phenotypic characteristics

## Abstract

In the family of Vespidae there are examples of social evolution that are particular, compared to those found among other social insects. The characteristics of eusociality are, however, found only in three subfamilies, those of Stenogastrinae, Vespinae and Polistinae, but the problem of whether eusociality appeared one or two times has long been debated. Biomolecular analysis studies have definitively demonstrated that the Stenogastrinae are the representatives of a social lineage completely independent from that of the Polistinae and the Vespinae. In the present paper, I want to emphasize the various phenotypic characteristics, especially studied by the group for the study of social insects of the University of Florence, which make these social wasps different from the others.

## Simple Summary

Eusociality is a biological condition that we find in only three subfamilies of the family Vespidae: that of the Polistinae, that of the Vespinae and that of the Stenogastrinae. Recently, molecular data have confirmed that the evolutionary lineage of the latter is independent from that of the other two. The present work reports the biological, physiological and behavioural characteristics of the eusociality of hover wasps provided in large part, in more than forty years of research, by the group of the University of Florence.

## 1. A Systematic Overview

**Figure 1:**
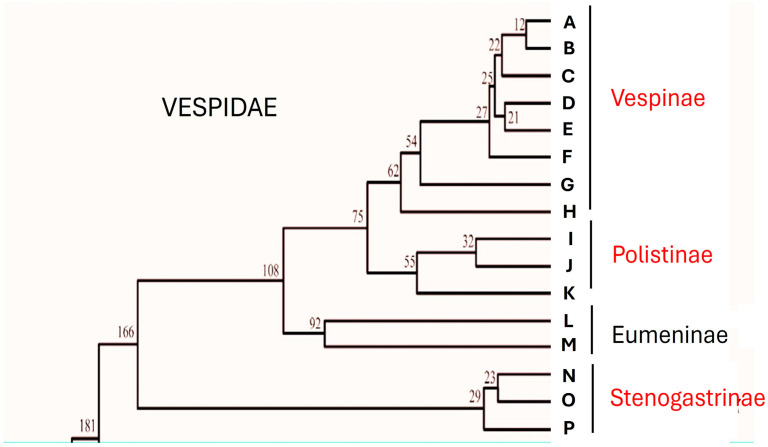
Simplified evolutionary timescale for the Vespidae inferred from mtgenome PCG12R datasets showing the relations between eusocial subfamilies (in red). Part of Figure 7 of Huang et al. [10] re-drawn. Capital letters correspond to the single species examined by these authors. (A) *Vespa velutina*; (B) *Vespa bicolor*; (C) *Vespa orientalis*; (D) *Vespa mandarinia*; (E) *Vespa ducalis*; (F) *Vespa affinis*; (G) *Vespula germanica*; (H) *Dolichovespula panda*; (I) *Polistes jokohamae*; (J) *Polistes humilis*; (K) *Parapolybia crocea*; (L) *Orancistrocerus aterrimus aterrimus*; (M) *Abispa ephippium*; (N) *Eustenogaster scitula*; (O) *Liostenogaster nitidipennis*; (P) *Parischnogater mellyi*. Numbers in the image are in Mya.

In the family Vespidae there are examples of social evolution that are particular compared to those found among other social insects. If, however, by eusociality we mean the presence of colonies in which there is overlapping of generations, common care of the offspring and, above all, reproductive division of labour among the members who form them [1], then these characteristics are found only in three subfamilies, those of Stenogastrinae, Vespinae and Polistinae. Although the evolutionary drive towards behaviours that result in increasingly complex forms of aggregation and care of offspring is a characteristic of the whole family, the problem of whether eusociality had appeared once or more times has long been debated. The cladogram provided by Carpenter in 1981 [2,3,4] proposed a progression of sociality starting from the Stenogastrinae (primitively eusocial) that continued in the Polistinae and Vespinae (highly eusocial). This interpretation, contested first by Schmitz and Moritz [5] and by Hines et al. [6], was then definitively changed by biomolecular analysis studies that showed that Stenogastrinae are the representatives of a social line completely independent from that of Polistinae and Vespinae (Bank et al. [7]; Peters et al. [8]; Piekarski et al. [9]; Huang et al. [10]; Luo et al. [11]) (Figure 1).

The research of the team from the Department of Biology of the University of Florence on these wasps, widespread in Southeast Asia, began together with Leo Pardi in 1979, with the first mission to the island of Java. The research continued until today with the contribution of various entomologists, chemists, geneticists, microbiologists of the same group. We have studied these wasps, especially with the eye of the naturalist, aiming to grasp the differences and similarities in those biological, physiological and behavioural traits present in part in the other two subfamilies as well. This comparison, and ultimately the structure of the two social lineages, is what I would like to summarize here, examining some of the main phenotypic characteristics (morphological, anatomical, physiological, and behavioural) of the Stenogastrinae colonies, the so-called “hover wasps” or “dragonfly wasps” due to their characteristic hovering flight. A review of this kind has already been presented by myself and various other authors [3,12,13,14,15,16] but allow me to propose it again, accompanied by more available data and personal reflections.

**The Stenogastrinae: genera and distribution.** A very recent paper counts a total of 76 described species [17]. The genera that make up the subfamily are seven: *Liostenogaster*, *Eustenogaster*, *Parischnogaster*, *Metischnogaster* and *Cochlischnogaster* live in the Eastern region from India to the Philippines, while the other two (*Stenogaster* and *Anischnogaster*) are limited to the Papuasian region [3,18]. The cladogram of the various genera (proposed by Carpenter and Starr [18]) has been partly revised by Huang et al. [10] (Figure 1). With the exception of the genus *Cochlischnogaster*, some species of the other genera have been studied (more or less thoroughly) by various groups, among which the Italian one has produced a considerable part of the works. They are forest dwellers, but some species also implant their nests on human structures. They form small societies and are not aggressive, despite having a venom apparatus. In short, “gentle” wasps are ideal for study by entomologists.

According to molecular analyses (the data of which could give too old estimates not yet supported by fossil records—James Carpenter personal communication), Stenogastrinae diverged from the group that led to other social wasps around 160 million years ago (Jurassic) while the division between today’s three major genera begins around 29 million years ago, in the Oligocene [10]. The distribution of the various groups is quite characteristic: the five genera of the eastern region seem to be well characterized by a centralization around the possible branch centre of the subfamily that is located in the middle of the Sunda. If the detachment of the subfamily from the other Vespidae occurred so early, the various genera have diversified in this region. We have no information on the timing of the differentiation of the genera except for *Eustenogaster*, *Liostenogaster* and *Parischnogaster,* which could date back to at least 29–36 million years ago [10,11]. *Stenogaster* and *Anishnogaster* may have diverged later, but since New Guinea was never united to Wallacea, their distribution may have been the result of more recent immigrations (already during the Pleistocene the distance between the Sunda and the Sehul was small due to the lowering of the sea induced by the last glaciation estimated at 25 to 15 million years ago) [19,20].

**Figure 2:**
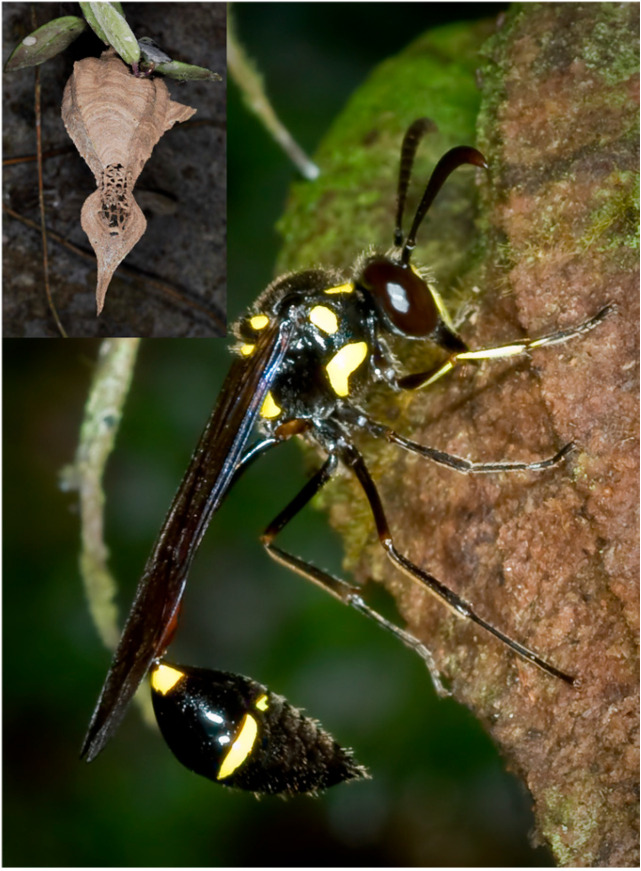
Female of *Eustenogaster fraterna* on her nest envelope. The entire nest can be seen at the left superior part of the picture.

**The main genera.** The genus *Eustenogaster* has the largest individuals of all the Stenogastrinae (Figure 2). It includes species that reach the northern limit of the subfamily range (with *E. nigra* able to overwinter [21]) and their colonies are formed by a number of females that reaches a maximum of six females in *E. fraternal* [22]. Nests with a particular architecture (in the shape of an inverted flask), more or less monotonous, are present in all the species described (Figure 2). They use abdominal secretion (see below) to a lesser extent than species of the other two genera and some species form ant guards (see later) in the early stages of nest building. Studies on the biology of three species are available (*E. calyptodoma* [23], *E. fraterna* [22,24], *E. nigra* [25,26], *E. eximia* [27]). From these we can deduce a sociality strongly limited by the architecture of the nursery and a very simplified social organization.

**Figure 3:**
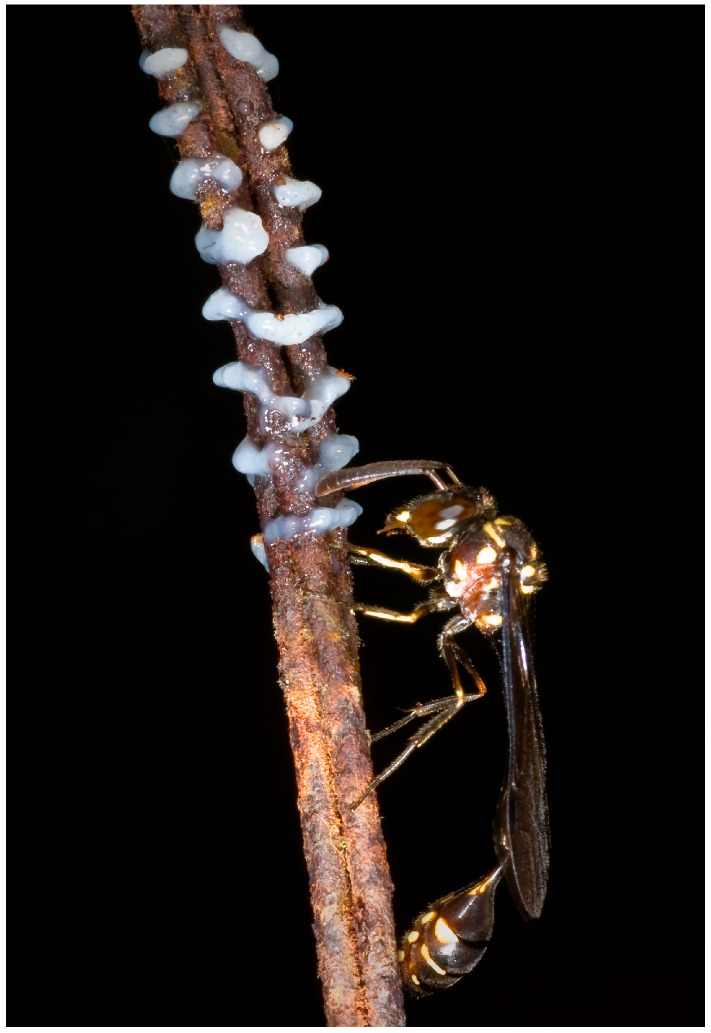
Female of *Parischnogaster jacobsoni* at the ant guard of her nest.

The genus *Parischnogaster* includes several species that are smaller than those of all other genera. They are characterized by dark coloration (Figure 3), and their nests are generally very camouflaged. *P. jacobsoni* [28], *P. mellyi* [29] and *P. nigricans serrei* [30] were the first species whose biology was studied in detail, with colonies consisting of a maximum of ten females. The nests have a simple architecture; the resemblance to lumps of earth characterizes most of the species but a group, which includes *P. striatula* and *P. alternata;* instead, it has nests with complex architecture [31,32], and the tendency (*P. alternata)* to form large agglomerations of colonies in suitable and defended places [33]. The colonies have a limited exchange of individuals, and the grouping offers a partial defence from predators (ants and hornets). Even in these cases, due to the particular architecture, the colonies are unable to accommodate a high number of females.

**Figure 4:**
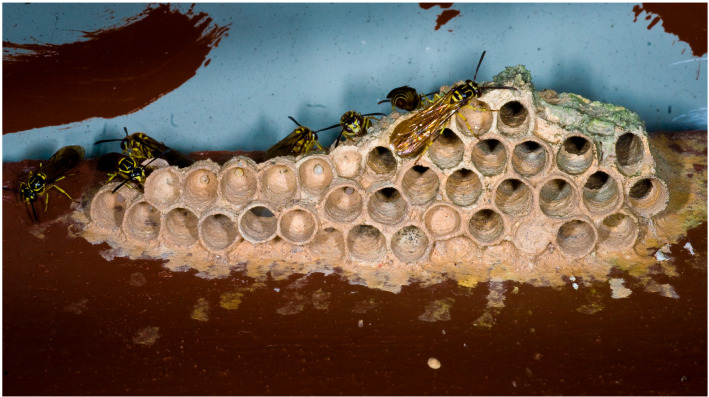
Colony of *Liostenogaster leonardi* with females and males. The nest is built with mud.

The genus *Liostenogaster* is certainly the most interesting and the most studied. It is basically made up of two groups of species: those that build mud nests (*L. flavolineata*, *L. pardii*, *L. leonardi*,, etc.) (Figure 4) and those that build nests with plant material (*L. vechti*, *L. tutua* etc.). In the first group, *L. flavolineata* can form large clusters of nests on the ceiling of caves or human buildings; in the second *L. vechti* gives rise to agglomerations of up to hundreds of nests arranged both horizontally and vertically on the horizontal and vertical surfaces of caves, walls and ceilings of buildings, etc. These two species have a nest architecture that allows them to place colonies close to each other that together can contain hundreds of individuals and that provide a partial defence from predators ensured, however, to the younger offspring also by an abundant supply of “pap” (see later). Nests arranged on thread-like substrates are provided with ant guards while species with nests provided with envelopes are found in *L. pardii* (mud envelope) [34] and in *L. abstrusa* (which builds multiple combs inside hollow twigs) [35].

**Morphology.** The morpho-anatomical differences in these wasps compared to the other social wasps (Polistinae and Vespinae) had already been noted by the first authors who saw the subfamily phylogenetically distant from the other social ones (Richards [36], van der Vecht [37], Spradbery [38]). They had highlighted that hover wasps had a particular morphology: very slender, with an extremely elongated peduncle, modest coloration in all genera. Wings, at rest, are not folded longitudinally like other social wasps. Stinging apparatus has a particularly shaped sting [14] and, when evaginated, it faces upwards (unlike the Polistinae and Vespinae). Another difference that is found in the morphology that differentiates the Polistinae + Vespinae from the Stenogastrinae is the presence in the former of the so-called “Van der Vecht organ”, a structure equipped with hairs present in the last sternite of the females in connection with integumentary glands, which is absent in the latter [14].

**Figure 5:**
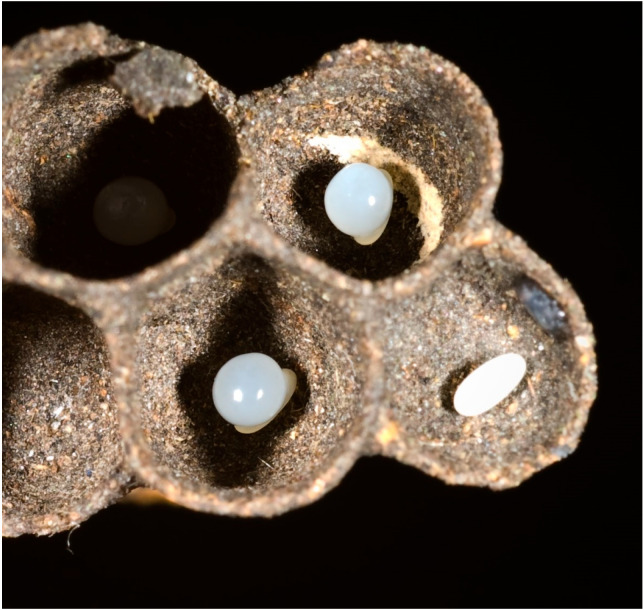
Particular of eggs with and without “pap”of *Parischnogaster mellyi*. The egg on the right had been deprived manually from the “pap”.

**Abdominal substance (the “pap”) and development.** The main differences with other social wasps are found in the development and care of immature offspring. Several authors had observed the presence of a gelatinous substance on eggs and small larvae [38,39,40,41,42] (Figure 5). The function of this substance (unique in all Vespidae) was considered trophic (hence the term “pap”) but, in reality, more accurate experiments, observations and analyses showed us a different function. The substance is produced by the Dufour gland and plays important roles in the biology of these wasps, starting with the laying of the egg in several stages described in detail [13,30,43] in *Parischnogaster nigricans serrei*. Various females of the colony can help to renew the supply of pap which later serves as a support for the larva at the time of egg hatching [43]. On it, the adults also place solid or liquid food until the larva, having become large, curls up, remaining in place inside the cell by pushing with its back. The adults at this point lay food inside its coils, food that can eventually be taken up for distribution to other larvae. When the larva is mature, it forms a pupa bent on itself. The adults seal, in some species, the cell with building material and then re-open it a few days later to remove the peritrophic sac emitted by the larva and re-close it; in some species the adults limit themselves to narrowing the walls of the cell. This breeding system is unique among all social wasps and is present, with few variations, in all the species of Stenogastrinae examined. *Anischnogaster laticeps* seems to be an exception to the rule [44]: in the nests of this wasp with a very limited sociality, we have not found pap balls on the eggs and larvae (but the substance can also act as a simple adhesive for the egg!) despite the presence in the females of a developed Dufour gland. There are four larval stages [29,45,46,47] or five [48]; however, the question does not seem to be definitively clarified. The need to use the secretion for various purposes has also influenced the morphology of the vulnerant apparatus and the release of venom [49] limiting its use for defence against predators. However, the venom is used as protection against pathogens [50].

Analyses of the composition of the substance have shown that it is composed mainly of hydrocarbons and some emulsifying compounds [51]; similar composition is found in the substance present in the Dufour gland and also in that used, in certain species, for the construction of barriers against ants (ant guards) ([52]; see also [40]) (Figure 3).

**The nest and its characteristics**. The nest represents a salient feature of social insects with a few exceptions. All species of the three subfamilies of eusocial wasps build shelters for raising offspring, and the architecture and construction techniques have similarities and differences. Compared to the Apidae, they do not have nests dug into the ground or nests built with materials, such as wax, produced independently but they rather use materials of various qualities obtained from the environment. The choice of these materials affects the structure and size of the nests and, of course, the size of the colonies as various authors had already observed [28,31,39,41,53,54]. However, the “cement”, the secretion that holds together the particles or fibres obtained for construction, is also important, excreted in both groups by the salivary glands. The resulting material is much more binding in Polistinae + Vespinae than that found in Stenogastrinae, and this clearly affects the size of the nests [55,56]. The architectural features of nests can influence biology [32]. In some species it should be noted that instead of plant material, mud is used in a clear reversal of the evolutionary trend (Figure 4). This phenomenon is found in both Polistinae + Vespinae (genus *Polybia*) and Stenogastrinae (genus *Liostenogaster*) and has probably evolved in response to the pressure exerted by particular predators (legionary ants in the first case and hornets in the second [55]).

Already the first observers of these wasps had noticed that the nests of the Stenogastrinae, unlike most of those of the Polistinae + Vespinae, lacked a peduncle. This was confirmed for all species of six genera studied. Some authors attribute the limited size of nests (and consequently those of societies) to this fact, given that the presence of a point attack can favour the defence of the nest and increase the possibility of making its construction independent of the attack substrate. Despite everything, the architecture of the nests of the Stenogastrinae has undergone an evolutionary radiation equal only to that presented by the Epiponini (which have lost the peduncle in some species).

**Figure 6:**
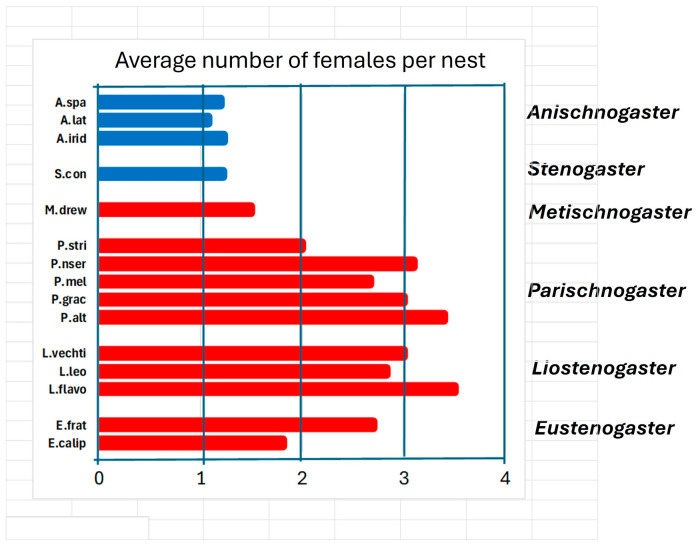
Average number of females present on nest reported for species of six genera of hover wasps: A. spa (*Anischnogaster* sp. [44]); A.lat (*A. laticeps* [44]); A.irid (*A. iridipennis* [57]). S. con (*Stenogaster concinna* [38]). M. drew (*Metischnogaster drewseni* [58]). P. stri (*Parischnogaster striatula* [59]), P. nser (*P. nigricans serrei* [45]). P. mel (*P. mellyi* [29]). P. grac (*P. gracilipes* [60]). P. alt (*P. alternata* [33]). L.vechti (*Liostenogaster vechti* [46]). L.leo (*L. leonardi* [47]). L.flavo (*L. flavolineata* [61,62]). E. frat (*Eustenogaster fraternal* [24]). E.calip (*E. calyptodoma* [23]).

**Social organization.** All species have at least one phase in their cycle in which the requirements for eusociality are met: overlapping of generations, division of labour, and common rearing of offspring. This phase in some species, however, can last very little, only a short stay with a mother who is the founder of the nest of a young daughter, who provides help of various kinds such as foraging, defending the nest and building it. In this case we should perhaps speak of extended parental care not unlike that of most social vertebrates. The young would find it advantageous to replace a mother who is close to death by inheriting the nest. This category includes all species belonging to the genera *Anischnogaster*, *Stenogaster* and *Metischnogaster*. In the species of these genera the size of the colonies is limited to a small number of individuals (Figure 6), and the average number of females present is less than two: the daughters remain to help their mother and then abandon her with the aim of founding their own nest.

In the most populous colonies of *Liostenogaster* and *Parischnogaster* the females present (which however rarely exceed 10 units) form dominance hierarchies (comparable to those found in *Polistes),* where the recognition of nestmates (based on chemical and visual characteristics) has been found and studied in various species [59,63,64,65,66].

The females of the lower ranks, however, retain the possibility of laying or abandoning their nests to join other colonies or to found their own nests. This behavioural plasticity is present in all species and defines well the primitively eusocial condition of the representatives of the subfamily. British researchers, using transcriptomics methods, have highlighted how the expression of genes that code for reproductive and non-reproductive behaviours can be influenced by hierarchical position and its possible changes [67]. Castes are therefore modelled in relation to the social environment and the behavioural plasticity that characterizes the females of the species of *Parischnogaster* and *Liostenogaster* is quite marked. The roles that females can assume are varied because the individuals who participate in the game are more numerous: the increase in individuals that find themselves on the nests grants more solutions to the subordinate females linked to the social situation (for example, the wait in the line of hierarchy of a colony to arrive at the egg-laying or the choice to participate in the rearing of more or less related offspring). This is the situation we find in most species even if in prolonged studies on *L. flavolineata* [61] and *P. nigricans serrei* [68] it was noted that, indeed, some individuals followed from the egg stage did not show any ovarian increase during their lifetime (*L. flavolineata*) or that there was a statistical difference between fertilized females (larger) and unfertilized females (smaller) (*P. nigricans serrei*). This could indicate, perhaps, an early pre-imaginal differentiation.

It has been experimentally seen by British researchers [67] that the disappearance of the dominant female (and currently the only egg layer) from a colony of *L. flavolineata* triggers changes at the gene level that affect the behaviour of other females. Unexpectedly, the genes that are activated are of particular interest to those that involve a greater investment in foraging efforts rather than those related to social rank. Comparing the results of a similar experimental situation in colonies of *Polistes* (Polistinae), the authors [69] realized that these indicate that a large part of the intracolonial transcriptomic differentiation in *L. flavolineata* is similar to that of *P. dominula* but does not offer much support for the existence of a genetic complex for sociality and that there is significant overlap of genes associated with foraging but not with reproduction.

**Figure 7:**
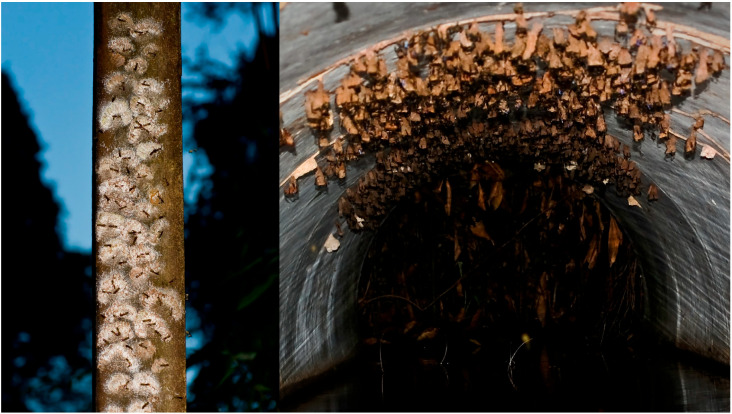
Clusters of colonies of *Liostenogaster vechti* (on the left) and of *Parischnogaster alternata.*

**Colony aggregates.** As we have seen, one of the characteristics of three species belonging to the genera *Liostenogaster* and *Parischnogaster* is the gathering of colonies in extremely small areas. In cases of *P. alternata* (Figure 7) and *L. flavolineata*, dozens of nests are found on flat and protected surfaces such as the ceilings of caves or human structures. In the case of *L. vechti*, the nests are instead aggregated on flat and vertical surfaces up to several hundreds (Figure 7).

We have studied these agglomerations from various points of view, and we have highlighted the purely defensive significance for the colonies that compose them, depending also on the architectural characteristics of the nests, against predators and parasitoids. In the case of *P. alternata* it is assumed that the defence is in particular directed towards hornets and ants, in *Liostenogaster* towards ants. In all cases, it is shown to be an advantage for colonies located in the centre of the agglomeration and a tendency of females to occupy or found nests in the central part [70,71]. It should be noted that these agglomerations are exclusively formed by three of the species that have the largest colonies both in terms of nest size and adult population, even if the colonies certainly appear independent of each other.

**Reproductive behaviour.** The information we have on this aspect of the biology of Stenogastrinae is quite scarce [14,53]. Mating has been observed a few times in *Parischnogaster*, both in the field and in captivity and has prominent characteristics among the various species. However, peculiar behaviour of males has allowed us to investigate how this is linked to the presence, in all genera, of groups of integumentary glands certainly involved in the production of pheromones [14]. These groups of glands are found in the gastral tergites of males and release their secretion on the outer surface. It is emblematic that even in the males of Polistinae and Vespinae there are similar groups of glands but that these are, instead, associated with gastral sternites. Reproductive behaviour has also been studied in captivity on *Parischnogaster mellyi* and the formation of both aerial territories (similar to those of *Metischnogaster* [53,72] and located in various points of the landscape marked with feces of both sexes has been highlighted [73].

## 2. Final Considerations

After this brief review of the biology of these wasps, also in the light of the most recent data collected on their phylogenetic position, we can ask ourselves some questions concerning, in particular, the origin of sociality in the group and its evolution:

1. What could have been the factors that favoured the origin of its sociality?
2. What were the factors that limited the size of the colonies?
3. What were the factors that prevented the evolution of sterile castes?

The situation of the various genera perhaps helps us to better understand how sociality developed in these wasps. It is highlighted that the genera *Liostenogaster* and *Parischnogaster* have the most social species if we take as a yardstick the size of the nests and the number of individuals present in various colonies (Figure 6). The genera *Eustenogaster* and *Metischnogasterr* (and probably *Cochlischnogaster*) appear to have been constrained in their development by a nest architecture that could not allow the growth of large colonies to maintain their camouflage characteristics [58]. The two Papuan genera present the most primitive sociality of all, right at the lower limit of the same.

The use of Dufour’s gland secretion is a common trait of all hover wasps (and therefore already present in the common ancestor) but there are differences between the various genera. We find large quantities of pap used in all the studied species of *Liostenogaster* and *Parischnogaster* while some species of *Eustenogaster* sometimes use it in a reduced way. *Metischnogaster* is poorly studied; *Stenogaster* and *Anischnogaster* have limited or almost no use of pap as in *A. laticeps.* I believe that the pap could therefore have been the factor that favoured the origin of eusociality in the first place and also its affirmation in the genera *Parischnogaster* and *Liostenogaster*, in particular the intensive use that this secretion is made in the rearing of offspring and in the stowage of food and its use in the defence of the youngest offspring and of the nest [43]. At the same time, this method of rearing the offspring, both from an energetic and temporal point of view (the development of immature offspring is around 100 days in the case of *Liostenogaster*), together with a very high mortality in the early larval stages, would have prevented the formation of large colonies [62].

If we take for granted that the invention and use of pap was a fundamental step in the origin of sociality, other causes have certainly influenced its evolution. Various authors have highlighted multiple factors that may have contributed to limiting its development, starting with the breeding method itself that is very difficult to implement in large quantities. Other factors such as the poor quality of the material used for the construction of the nest and the lack of the peduncle, the restrain of the nest size which increases their camouflage characteristics due to the need for defence against hornets, the lack of development of a social defence against vertebrate predators based on venom, the long period of development of immature offspring compared to the average life span of adults, and parasitism (especially of Diptera Tachinidae in the case of *Anischnogaster* [57,74]) would have had a negative effect on the development of sociality. Impediments of the first type have certainly blocked species of the genus *Metischnogaster,* strong parasitic pressures of the genera *Anischnogaster* and *Stenogaster.*

Some genera have tried to overcome these limits without being able to achieve those social characteristics similar to those of Polistinae + Vespinae. The architecture of the nest also has a special influence on the construction of agglomerates in the three species mentioned above and therefore on the reunion of many individuals even from different colonies. However, although exchanges of nests of some females have been reported (also in another species, *Parischnogaster striatula*), no behaviours have been observed that could indicate any organization at a higher level than the colonial one. A particular case is represented by *L.topografica* which builds large (or rather, extensive) nests on flat substrates [75]. The architecture of these nests consists of cells thickened along ribs that branch off from a centre and are covered with a fine red powder that has been shown to be repellent to ants. Colonies group together a large number of individuals, but the nests are, however, isolated. One could think of a further evolution of the nests of *L. vechti* and attempt to establish more numerous colonies similar to those of Polistinae + Vespinae through an aggregation route to sociality but this, at the moment, seems only hypothetical.

These data highlight the peculiar social evolution of Stenogastrinae wasps compared to that followed by the other two subfamilies and, even if they have not reached a high degree of sociality comparable to that of the latter, such as the creation of large colonies and the division of individuals into pre-imaginal castes, these insects represent an independent and original evolutionary line towards what is called eusociality. The development in some genera of complex behaviours that regulate the social life of small groups of individuals such as dominance hierarchies, the recognition of colonial mates, the chemical defence against pathogens and the participation of various individuals in the rearing of immature offspring are convergent characters with the highly eusocial wasps.

## Data Availability

No new data were created or analyzed in this study.
